# Local Somatodendritic Translation and Hyperphosphorylation of Tau Protein Triggered by AMPA and NMDA Receptor Stimulation

**DOI:** 10.1016/j.ebiom.2017.05.012

**Published:** 2017-05-17

**Authors:** Shunsuke Kobayashi, Toru Tanaka, Yoshiyuki Soeda, Osborne F.X. Almeida, Akihiko Takashima

**Affiliations:** a Department of Biochemistry, School of Pharmacy, Nihon University, 7-7-1, Narashinodai, Funabashi, Chiba 274-8555, Japan; b Clinical Research Center, Fukushima Medical University, 1 Hikarigaoka, Fukushima 960-1295 Fukushima, Japan; c Max Planck Institute of Psychiatry, Kraeplinstrasse 2-10, 80804 Munich, Germany; d Laboratory for Alzheimer's Disease, Department of Life Science, Faculty of Science, Gakushuin University, 1-5-1 Mejiro, Toshima-ku, 171-8588, Tokyo, Japan

**Keywords:** Somatodendritic localization of tau, Tau mRNA, RNP particle, Local translation, AMPA and NMDA receptors

## Abstract

Tau is a major component of the neurofibrillary tangles (NFT) that represent a pathological hallmark of Alzheimer's disease (AD). Although generally considered an axonal protein, Tau is found in the somato-dendritic compartment of degenerating neurons and this redistribution is thought to be a trigger of neurodegeneration in AD. Here, we show the presence of tau mRNA in a dendritic ribonucleoprotein (RNP) complex that includes Ca2^+^-calmodulin dependent protein kinase (CaMK)IIα mRNA and that is translated locally in response to glutamate stimulation. Further, we show that Tau mRNA is a component of mRNP granules that contain RNA-binding proteins, and that it interacts with Myosin Va, a postsynaptic motor protein; these findings suggest that tau mRNA is transported into dendritic spines. We also report that tau mRNA localized in the somato-dendritic component of primary hippocampal cells and that a sub-toxic concentration of glutamate enhances local translation and hyperphosphorylation of tau, effects that are blocked by the gluatamatergic antagonists MK801 and NBQX. These data thus demonstrate that alpha-amino-3-hydroxy-5-methyl-4-isoxazole propionic acid (AMPA) and *N*-methyl-d-aspartate (NMDA) stimulation redistributes tau to the somato-dendritic region of neurons where it may trigger neurodegeneration.

## Highlights

• Tau mRNA binds to Staufen1- and FMRP-containing ribonucleoprotein (RNP) particles in dendrites and synapse.
• AMPA and NMDA receptor-mediated glutamatergic stimulation triggers local translation of tau protein.
• Over stimulation of AMPA and NMDA receptors is a cause of somatodendritic localization of tau found in degenerating neurons.

We show here that glutamate-activated AMPA and NMDA receptors regulate local translation of tau protein and recruit mechanisms that lead to the accumulation of hyperphosphorylated tau and enhance the neurofibrillary lesions. Glutamate stimulation is thought to cause neurodegeneration in AD; memantine, which relieves the aberrant excitation of glutamate on NMDA receptor, has efficacy in AD therapy. Aβ, stroke or head trauma, which are known as higher risk of AD in etiological studies, evoke an exaggerate NMDA receptor stimulation. Therefore, our conclusion possibly provides a common mechanism for causing neurodegeneration in AD.

## Graphical Abstract

**Figure:**
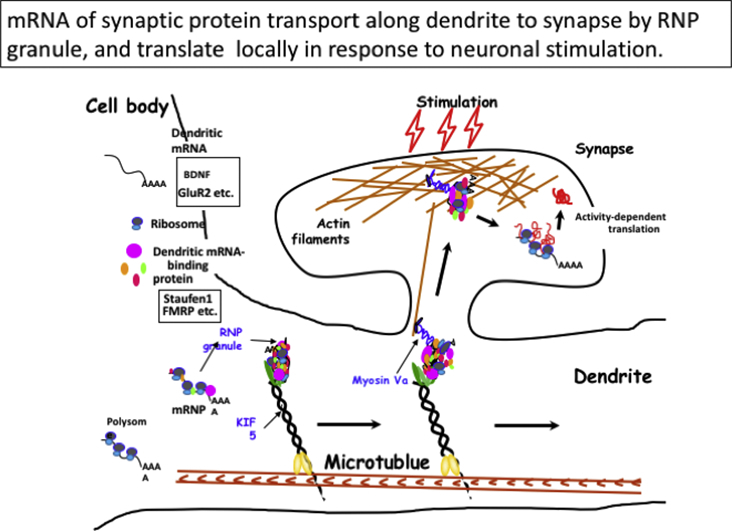

## 1 Introduction

Intracellular inclusions of hyperphosphorylated tau protein (neurofibrillary tangles, NFT) and extracellular deposits of amyloid β (Aβ) are prominent neuropathological features in Alzheimer disease brains. The propagation of NFTs from the entorhinal cortex to the neocortex, followed by neuron and synapse loss, corresponds closely with the clinical progression of Alzheimer's disease (AD) signs, from impaired memory to dementia; this suggests that the formation and propagation of NFT may be involved in the symptomatology of AD.

In the healthy brain, tau is an exclusively axonal protein, engaged in the assembly and stability of microtubules. In contrast, in the AD brain, tau is hyperphosphorylated and forms fibrils that appear as neuropil threads in dendrites and as NFTs in the somatodendritic compartment and axons. NFT formation is preceded by a pre-tangle stage where non-fibrillar and hyperphosphorylated tau accumulates in the soma and dendrites of neurons (Uchihara et al., 2001, Götz et al., 1995). These observations indicate that tau hyperphosphorylation in somatodendrites precedes tau fibrillation and the appearance of neurofibrilar lesions. Thus, understanding the mechanism of tau missorting is an indispensible prerequisite for the development of therapeutic interventions against AD and other tauopathies.

Various mechanisms of tau transport into the somatodendritic compartment have been proposed. For example, while retrograde movement of tau is prevented by a barrier at the initial segment of the axon, hyperphosphorylated tau can transgress this barrier and redistribute to other parts of the neuron (Li et al., 2011). Besides the recent description that amyloid β (Aβ) oligomers induce hyperphosphorylated tau missorting into hippocampal dendrites (Zempel et al., 2013), little is known regarding the pathophysiological stimuli that lead to the accumulation of hyperphosphorylated tau in dendrites.

Recent studies have ascribed a role for tau in the physiological regulation of synaptic function. For example, Ittner et al. reported that tau is required for Fyn-mediated *N*-methyl-d-aspartate (NMDA) receptor activation in the postsynaptic density (PSD) (Ittner et al., 2010), and tau has been shown to be essential for the induction of long term depression (LTD) (Kimura et al., 2013) as well as Brain-derived neurotrophic factor (BDNF)-dependent morphological plasticity (Chen et al., 2012). Other studies demonstrated that neuronal activation leads to the translation of proteins that participate in synapse formation and plasticity (*e.g.* Calcium/calmodulin dependent protein kinase II: CaMKIIα, Glutamate receptor: GluR, and Arc) in dendritic spines (Bramham and Wells, 2007, Steward and Halpain, 1999, Steward and Worley, 2002, Steward and Schuman, 2003); the mRNAs encoding these molecules are carried to the dendrite by messenger ribonucleoprotein (mRNP) granules, a complex of mRNAs and mRNA-binding proteins. Given this, and evidence for the involvement of tau in the modulation of synaptic function (Kimura et al., 2013), we hypothesized that tau mRNA is transported into dendrites and spines and locally translated upon stimulation as well as the other synaptic proteins. We here verified this hypothesis by demonstrating that stimulation of glutamatergic receptors results in the transport of tau mRNA to dendrites where it is subsequently translated.

## 2 Materials and Methods

### 2.1 Animal Studies

Animal studies were performed under the Guidelines for Animal Experiments at Nihon University.

### 2.2 Antibodies and Reagents

Anti-tau (rabbit) (Cat# sc-1996 RRID:AB_632468), anti-Microtubule associate protein 2 (MAP2) (rabbit) (Cat# sc-20172 RRID:AB_2250101) and anti-β-actin (rabbit) (Cat# sc-10731 RRID:AB_2223515) antibodies were from Santa Cruz Biotechnology. Rabbit anti-Staufen1 (Cat# ab73478 RRID:AB_1641030) and mouse anti-MAP2 (Cat# ab11267 RRID:AB_297885) antibodies were from Abcam. Anti-Fragile X mental retardation protein (FMRP) (Cat# MAB2160 RRID:AB_2283007) and anti-tau1 (Cat# MAB3420 RRID:AB_94855) antibodies (both from mouse) were from Millipore. Mouse anti-AT8 antibody (Cat# MN1020 RRID:AB_223647) was purchased from Thermo Scientific. Anti-Myosin Va (Cat# 3402S RRID:AB_2148475) and anti-S6 antibodies (Cat# 2217 also 2217L, 2217S RRID:AB_331355) (both raise in rabbit) were from Cell Signaling Technology. Alexa Fluor 488-conjugated goat anti-mouse IgG (Cat# A-11001 RRID:AB_2534069), Alexa Fluor 488-conjugated goat anti-rabbit IgG (Cat# A-11034 also A11034 RRID:AB_2576217), Alexa Fluor 555-conjugated goat anti-mouse IgG (Cat# A-21422 also A21422 RRID:AB_141822), and Alexa Fluor 555-conjugated goat anti-rabbit IgG (Cat# A-21428 also A21428 RRID:AB_141784) were purchased from Thermo Fisher Scientific. Glutamate, 4′,6-diamidino-2-phenylindole (DAPI), cycloheximide, 2,3-dioxo-6-nitro-1,2,3,4-tetrahydrobenzo[*f*]quinoxaline-7-sulfonamide (NBQX), and D-(-)-2-amino-5-phosphonopentanoic acid (D-AP5) were obtained from Wako Pure Chemical Industries. MK801 and (*S*)-3,5-dihydroxyphenylglycine (DHPG) were purchased from Sigma-Aldrich. 2-chloro-4-((2,5-dimethyl-1-(4-(trifluoromethoxy) phenyl)-1H- imidazol-4-yl)ethynyl)pyridine (CTEP) was purchased from Selleck Chemicals. Fluorescein 12-UTP and T7 RNA polymerase were purchased from Roche. Alkaline phosphatase (Calf intestine) was from Toyobo.

### 2.3 Immunoprecipitation, Western Blotting and RT-PCR

Immunoprecipitation was carried out using Dynabeads Protein G (Life Technologies) following the manufacturer's protocol. Antibodies (2 μg of each) were bound to the beads and incubated with cell extracts, cell fractions or sucrose gradient fractions (4 h, 4 °C). Beads were recovered, washed with PBS containing 0.1% BSA, and the immune complex eluted (20 mM Tris–HCl [pH 7.5], 140 mM NaCl, and 2% SDS). Proteins were analyzed by Western blotting and co-immunoprecipitated mRNAs were extracted for use as templates in RT-PCR assays. For Western blot analysis, proteins were separated on 10% SDS-PAGE gels, transferred to polyvinylidene difluoride (PVDF) membranes and incubated with relevant primary antibody and corresponding secondary horseradish peroxidase-conjugated antibody (GE Healthcare Life Sciences); protein signals were detected using Enhanced Chemi Luminescence (ECL) (GE Healthcare Life Sciences) and semi-quantified by densitometry. Where necessary, antibodies were stripped using Restore PLUS Western blot stripping buffer (Thermo Scientific).

For RT-PCR, RNAs were extracted with SDS-phenol-chloroform and dissolved in water. First-strand cDNA was synthesized with Moloney Murine Leukemia Virus (MMLV) reverse transcriptase (Takara) using an oligo (dT) primer; double-strand cDNA was synthesized using specific primer pairs (Supplementary Table 1). The RT-PCR products were stained with ethidium bromide and analyzed using a gel documentation system (BioRad GelDoc XR Plus ImageLab).

### 2.4 Preparation of Synaptosomal Fractions

Synaptosomal fractions were prepared from cerebral cortical and hippocampal tissues from 5-week old male CB57BL/6J mice (Charles River Laboratories International, Inc.). All solutions used contained 1 mM MgSO_4_, 15 mM *N*-tris[hydroxymethyl]-methyl-2-aminoethanesulfonic acid (Na-TES, pH 7.4), RNase inhibitor (0.2 unit/μl, Takara) and protein inhibitor (complete cocktail without EDTA, Roche). After homogenization of tissues in 9 volumes of 0.3 M sucrose solution (first with a loose-type, then with a tight-type Dounce homogenizer; Wheaton), homogenates were passed through a series of nylon mesh filters (111, 70 and 52 μm; Spectrum) and centrifuged (900 ×* g*, 10 min, 4 °C). After washing with 0.3 M sucrose, pellets were suspended in 18% Ficoll/0.3 M sucrose solution and centrifuged (7500 ×* g*, 40 min, 4 °C, Beckman Coulter SW40Ti rotor). Supernatants were then diluted with 2 volumes of the Ficoll/sucrose solution and centrifuged (13,000 ×* g*, 20 min, 4 °C SW40Ti rotor) and the resulting pellet was resuspended in 0.3 M sucrose solution containing 1% NP-40 to yield synaptosomal fractions. Proteins and RNAs were analyzed by Western blotting and RT-PCR, respectively. When glutamate treatments were used, primary culture of E18 CB57BL/6J mouse hippocampal neurons were grown on polylysine-coated 5-cm dishes (Iwaki) and maintained for 16 days *in vitro*. Synaptosmal preparations were prepared after treatment of cells with glutamate.

### 2.5 Preparation of Neuronal mRNP Granule-enriched Fractions

Cerebral cortices and hippocampi from 34-week old wild-type and tau-knockout CB57BL/6J mice were prepared by the Institute of Immunology, Utsunomiya (Japan). Briefly, brains were washed with ice-cold PBS and homogenized in TKM buffer (50 mM triethanolamine [pH 7.8], 50 mM KCl, 5 mM MgCl_2_, 0.25 M sucrose, 1 mM PMSF, protein inhibitor [complete cocktail without EDTA, Roche], 1 mM DTT and RNase inhibitor [0.2 unit/μl, Takara]). Homogenates were centrifuged (1000 ×* g*, 10 min, 4 °C) and 0.5 ml of each supernatant was loaded onto a 15–45% sucrose gradient (9 ml) with a 0.75 ml cushion (45% sucrose) before centrifugation at 36,000 rpm (2 h, 4 °C, Beckman Coulter SW40Ti rotor). The gradient was fractionated, and the distribution of RNAs and ribosomal protein in each fraction was monitored by absorbance at 254 nm and Western blotting of S6 ribosome protein, respectively.

### 2.6 Primary Culture, Glutamate Treatment and Immunocytochemistry

Hippocampal neurons were prepared from embryonic (E18) CB57BL/6J mice. Cells were grown in polylysine-coated glass chambers (Lab-Tek) and maintained for 16–17 days in neurobasal medium containing 0.5 mM l-glutamine and 2% B27 Supplement (Gibco). When differentiated, neurons were exposed to glutamate (0.5 mM) for 5 min and the medium was promptly replaced with fresh neurobasal medium and incubated for a further 25 min, before fixation with 4% paraformaldehyde (15 min, room temperature), permeabilization (1% Triton-X100), and blocking with DMEM containing fetal bovine serum. Specimens were then incubated with appropriate primary antibody (2 h, RT). After washing with PBS, specimens were incubated with corresponding secondary antibody (Alexa Fluor 488-conjugated goat anti-rabbit antibody, Alexa Fluor 555-conjugated goat anti-mouse antibody, Alexa Fluor 488-conjugated goat anti-mouse antibody, or Alexa Fluor 555-conjugated goat anti-rabbit antibody) for 1 h at RT. Preparations were examined using an Olympus IX70 inverted microscope linked to a DP-70 imaging system or a Zeiss LSM-710 fluorescence laser-scanning confocal microscope, as appropriate.

### 2.7 Preparation of RNA Probes

mRNA was purified from a CB56BL/6J mouse brain using NucleoSpin RNA and NucleoTrap mRNA Mini Kit (Takara), after which two double-stranded fragments of 232 and 231 nucleotides of mouse tau mRNA were synthesized by RT-PCR using appropriate primer pairs:

**5′-non-coding region:** Forward, 5-CGGCCTCCAGAACGCGCTTTCTCGGCCGCGCG-3′

Reverse, 5′-TAATACGACTCACTATAGGGATCTCCAGCATGGTCTTCCATTG-3′

**3′-non-coding region:** Forward, 5′-CTTCTTCCTCCCTCCCCGCAAGGTGGGAGGTCCTGAGCG-3′

Reverse; 5′-TAATACGACTCACTATAGGGCCCTCCCACTACAGAAGTGCCAGA-3′

The probe RNA was prepared in accordance with the manufacturer's instructions for RNA labeling with Fluorescein 12-UTP by *in vitro* transcription with T7 RNA polymerase (Roche).

### 2.8 *In situ* Hybridization

Hippocampal neurons were fixed and permeabilized as described above before washing with PBS. Prehybridization was performed with Probe Diluent (GenoStaff) containing 500 μg/ml heat-denatured salmon sperm DNA (1 h, 50 °C in a humidified incubator). RNA probes were diluted with Probe Diluent to 1 μg/ml, denatured (15 min, 95 °C) and immediately placed on ice. Hybridization was carried out over 20 h at 50 °C, after which specimens were washed (10 min at 50 °C), once with 1 × HybriWash (GenoStaff), once with 1 × HybriWash containing 50% formamide, twice with 1 × HybriWash, and twice with 0.1 × HybriWash. The fluorescein-labeled tau mRNA was visualized using an Olympus IX70 inverted microscope linked to a DP-70 imaging system. For immunostaining, hybridized cells were first blocked with DMEM/10% fetal bovine serum (30 min).

## 3 Results

### 3.1 RNP Granules Containing Tau mRNA are Detectable in Synaptosomal Fractions

**Fig. 1:**
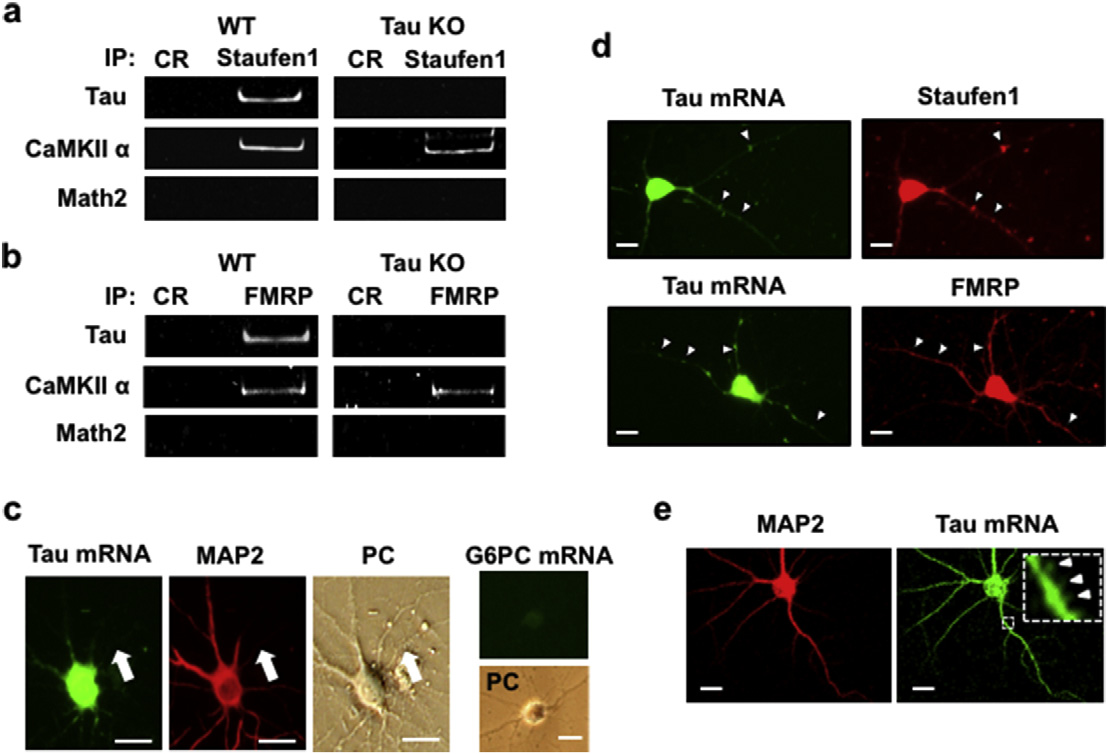
Tau mRNA is a constituent of FMRP- and Staufen1-positive dendritic RNP granules. Tau and CaMKIIα mRNAs detectable in (a) Staufen1- and (b) FMRP-positive immunoprecipitates from RNP granules (fraction 13; Supplemental Fig. 1) from hippocampi of wild type (WT), but not tau knockout (KO), mice. The two images on the *left* of panel c show tau mRNA in a neuron (fluorescence *in situ* hybridization: Alexa Fluor 488) whose dendrites are MAP2-immunostained (Alexa Fluor 555); a phase contrast (PC) image (*third-from-left*) and a negative control (G6PC mRNA, *fourth-from-left*) are also shown. Liver- and kidney-specific G6PC mRNA was not detected. *Arrows* indicate axons. Panel (d) shows Staufen1- and FMRP-immunopositive neurons that co-express tau mRNA. *Arrowheads* indicate granule structures in dendrites expressing tau mRNA and Satufen1 or FMRP. Panel (e) shows the neuronal distribution of tau mRNA as revealed by confocal microscopy. *Arrowheads* (inset) indicate dendritic spines. Scale bar: 10 μm.

Since ribonucleoprotein (RNP) granules, containing the mRNA-binding proteins Staufen1 and FMRP, are important for the transport of mRNA species to dendrites and their translation therein (Kiebler et al., 1999, Köhrmann et al., 1999, Greenough et al., 2001, Zalfa et al., 2006), we first purified RNP granules from the hippocampi of both wildtype (WT) and tau knockout (KO) mice (Supplemental Fig. 1). Whereas RNP granules recovered by immunopreciptation with anti-Staufen 1 (Fig. 1a) and anti-FMRP (Fig. 1b) from the hippocampi of wild type (WT) and tau knockout (KO) mice expressed CamKIIα and tau mRNA, immunoprecipitates from tau KO animals only contained CamKIIα mRNA; importantly, Math2 mRNA, whose expression is restricted to the cytoplasm, did not co-immunpreicipate with the RNPs in preparations from WT and tau KO mice (Fig. 1a, b), indicating that tau is bound to Staufen1- and FMRP-containing mRNP in dendrites. Confirming this, *in situ* hybridization analyses demonstrated that tau mRNA can only be visualized in cell bodies and MAP2-positive neurites (Fig. 1c).

Further, immunostaining showed that tau mRNA co-localizes with the mRNA-binding proteins Staufen1 and FMRP and that the colocalization occurred within a granular structure, most likely mRNP granules (Fig. 1d). Since tau mRNA was also detectable in dendritic spines (Fig. 1e; arrow head), we analyzed PSD95-positive synaptosomal fractions from 5 week-old wild type mice; these were found to contain tau, CamKIIα and BC1 RNA (dendritic small non-coding RNA) (Kobayashi et al., 1991, Tiedge et al., 1991), but not the transcription factor Math2 (Supplemental Fig. 2a). Moreover, immunoprecipitation of hippocampal synaptosomes demonstrated that BC1 RNA and the mRNAs encoding CaMKIIα and tau are associated with Staufen1, FMRP and myosin Va (Supplemental Fig. 2b). The latter is a postsynaptic motor protein that is likely involved in the trafficking of Staufen1- and FMRP-tau mRNA complexes to the postsynapse (Balasanyan and Arnold, 2014, Yoshimura et al., 2006). Similar results were observed when synaptosomal preparations from 34 week-old mice were examined (Supplemental Fig. 2c). Collectively, this set of results shows that tau mRNA, complexed to Staufen1- and FMRP-containing RNP granules, is present in dendrites and post-synaptic sites.

### 3.2 Stimulation of AMPA and NMDA Receptors Triggers the Local Tau mRNA Translation in Dendrites

**Fig. 2:**
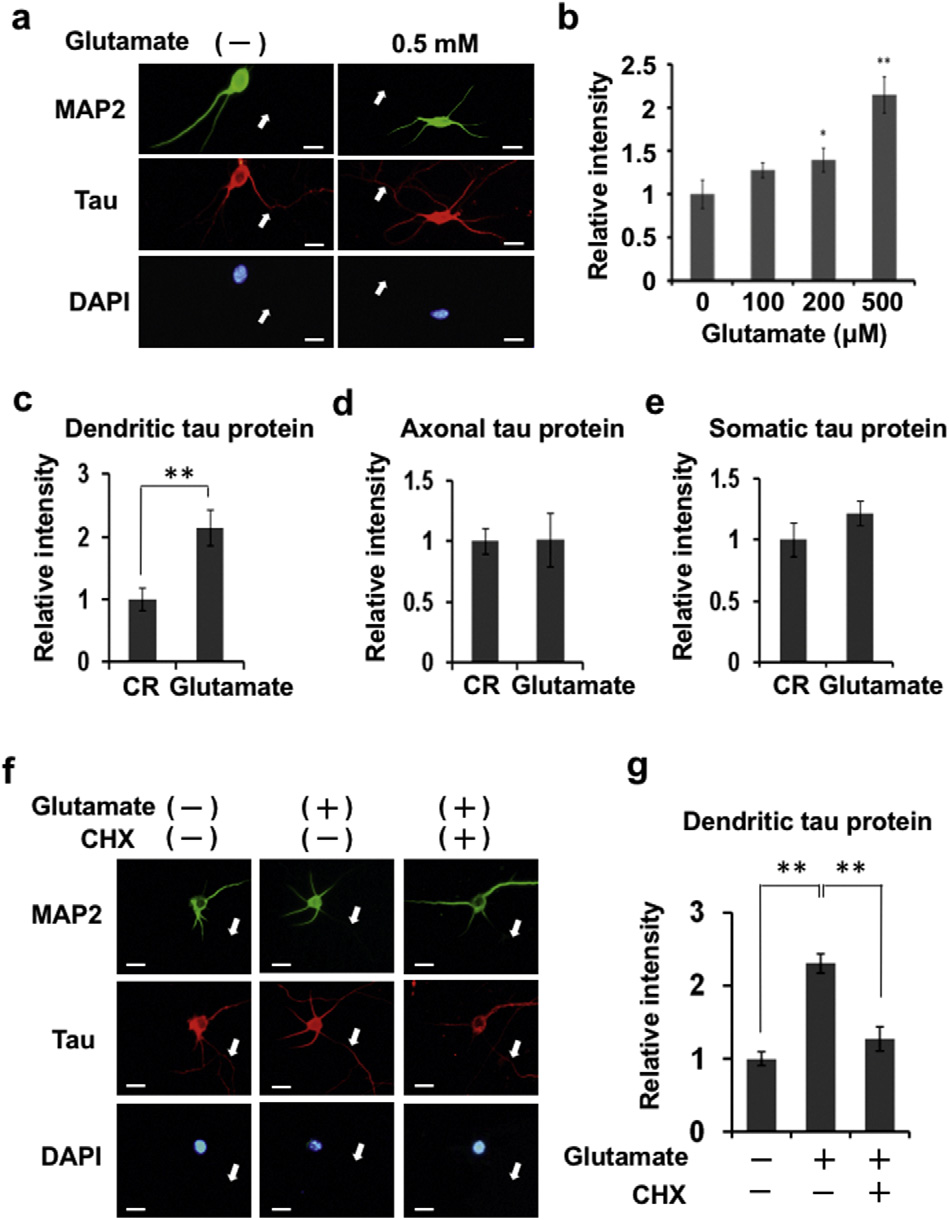
Glutamate stimulates local translation of tau protein from RNP granules in dendrites. Tau protein immunoreactivity was observed 25 min after brief (5 min) application of glutamate (0–0.5 mM) to hippocampal cultures (a-e). Quantitative analysis of tau (Alexa Fluor 555) immunoreactivity is shown in panels (b-e) as relative tau protein levels (fluorescence intensity) in dendrites (c), axons (d) and soma (e) in glutamate-treated and untreated control (CR) hippocampal neurons; for this, approximately 30 neurons were evaluated. Data, expressed as mean ± SEM, were normalized to intensity of nuclear DAPI staining. **P < 0.01 (Student's *t*-test). Scale bar: 10 μm. To demonstrate that glutamate triggers translational elongation in dendrites, primary hippocampal neurons were treated with glutamate (0.5 mM, 5 min) with or without cycloheximide (CHX, 20 μg/ml). Tau protein expression was rapidly upregulated (within 25 min of glutamate application) in MAP2-immunopositive dendrites of cells, and effect blocked by CHX (f). To quantify levels of tau protein, signal intensities obtained for approximately 30 dendrites (glutamate-treated or glutamate/CHX-treated neurons) using the NIH ImageJ software package were normalized to fluorescence intensity of each corresponding DAPI-stained neuron. Data represent means and standard errors. **P < 0.01 *versus* control or in presence of CHX (Student's *t*-test). Scale bar: 10 μm.

The translation of mRNA in synapses depends on neuronal activity (St Johnston, 2005, Krichevsky and Kosik, 2001). Double-immunostaining using anti-tau and anti-MAP2 antibodies (Fig. 2a) revealed tau protein localization in soma and MAP2-negative neurites (axons), while there is a faint tau immunoreactivity in MAP2-positive neurites (dendrites). Brief exposure of hippocampal neurons to glutamate (0.5 mM, 5 min) enhanced tau immunoreactivity in MAP2-positive dendrites, but not in cell bodies and axons (Fig. 2a). Quantitative analysis of instensity of tau immunoreactivity in dendrites (Fig. 2c), axons (Fig. 2d), and soma (Fig. 2e), after normalization to fluorescent intensity of DAPI (labels DNA), revealed that brief neuronal activation with glutamate modulates dendritic levels of tau protein in a dose-dependent manner (Fig. 2b). Further analysis showed that a sub-toxic dose of glutamate (0.5 mM, 5 min) causes a doubling of tau protein levels in dendrites (Fig. 2c), without a parallel increase in the amount of dendritic tau mRNA (Supplemental Fig. 3) or alterations in tau protein expression in axons and soma (Fig. 2d, e). Lastly, the glutamate-induced changes in dendritic tau protein levels were abolished when neurons were co-incubated with cycloheximide (Fig. 2f, g), indicating that glutamate stimulates rapid local translation of tau mRNA in dendrites.

**Fig. 3:**
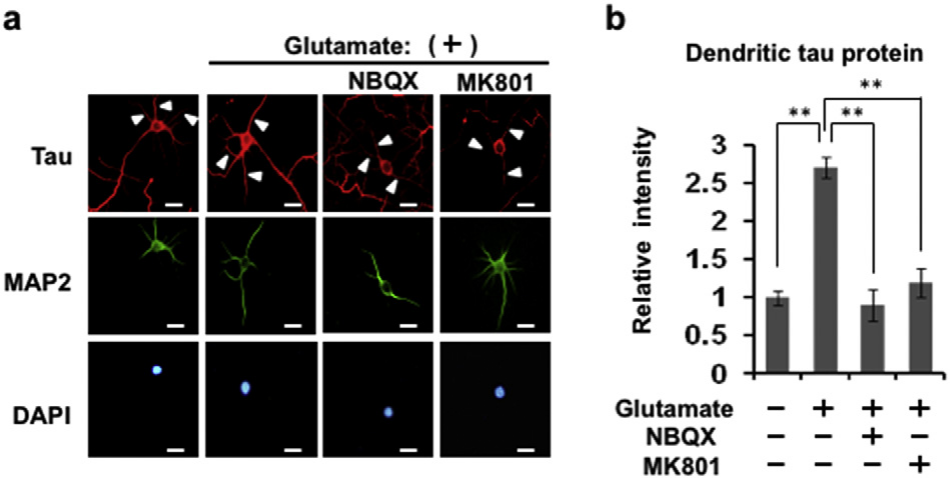
AMPA and NMDA receptor activation stimulates local translation of tau protein in dendrites. Glutamate (0.5 mM) effects were blocked by NBQX (50 μM) and MK801 (10 μM), the respective pharmacological antagonists of AMPA and NMDA receptors (a). Quantitative analysis of tau (Alexa Fluor 555) immunoreactivity is shown in panel (b) after normalization to tau protein levels (fluorescence intensity) in dendrites in glutamate-treated and untreated control hippocampal neurons; for this, approximately 80 neurons were evaluated. Data, expressed as mean ± SEM **P < 0.01 (Student's *t*-test). Scale bar: 10 μm.

Glutamate is a prominent excitatory transmitter whose actions are mediated by ionotropic (such as NMDA, AMPA) and metabotropic glutamate receptors; glutamatergic mechanisms are implicated in neurodegenerative processes triggered by various risk factors (Parsons and Raymond, 2014). Experiments in hippocampal cultures ruled out a role for metabotropic receptors (Supplemental Fig. 4). On the other hand, results obtained after antagonizing either AMPA or NMDA receptors with NBQX, MK801(Fig. 3a, b), or D-AP5 (Supplemental Fig. 5), demonstrated that glutamate induces the rapid translation of tau mRNA in dendrites by activating AMPA and NMDA receptors.

### 3.3 Stimulation of AMPA and NMDA Receptors Causes the Somatodendritic Localization of Phosphorylated Tau

**Fig. 4:**
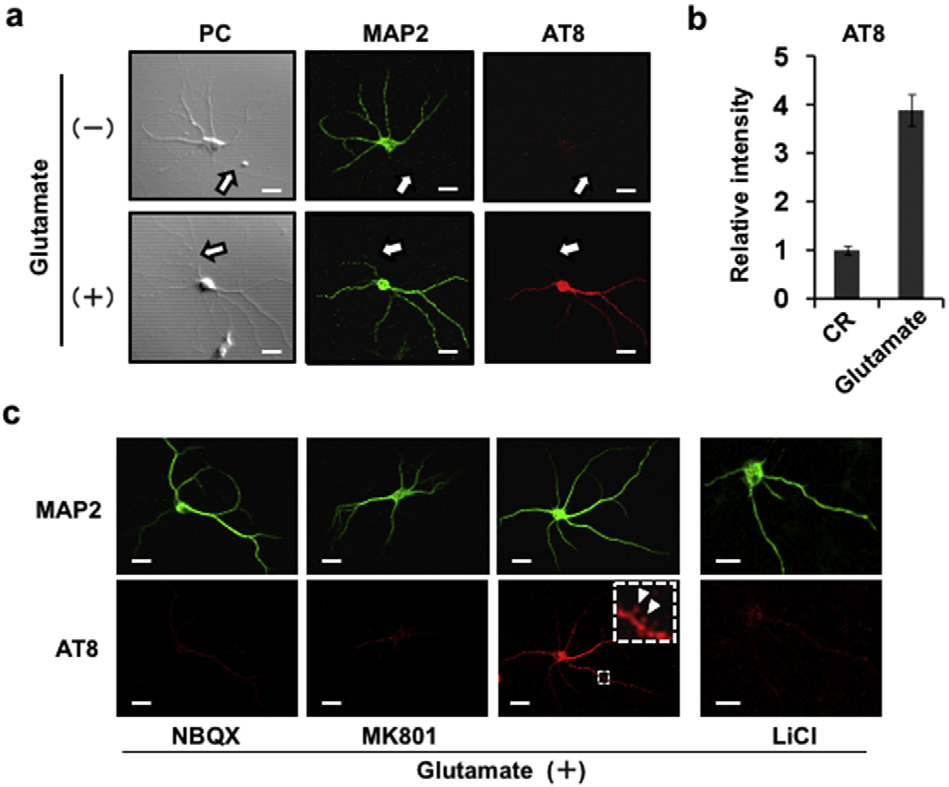
Glutamate induces mis-localization of phosphorylated tau to somatodendrites. Differentiated hippocampal neurons were exposed to glutamate (0.5 mM, 5 min) and, after 25 min, immunostained using antibodies against MAP2 (visualized with Alexa Fluor 488) and AT8 (Alexa Fluor 555) (a); semi-quantitative levels of phospho-tau (AT8) were derived by normalizing signal intensities to those obtained in untreated control (CR) cells (b). *PC*, phase-contrast images of corresponding cells; *arrows*, axon; *scale bar*, 10 μm. Glutamate effects were lost in neurons exposed to the AMPA and NMDA receptor antagonists NBQX (50 μM) and MK801(10 μM) or to LiCl (inhibitor of GSK-3β, 5 mM) (c). Inset shows magnified image of AT8-positive dendritic spines (white arrowheads).

Intraneuronal inclusions of hyperphosphorylated tau protein (neurofibrillary tangles, NFT), a common neuropathological finding in AD, are associated with neuronal and synapse loss and correlate with the extent of symptomology in patients (Uchihara et al., 2001) and animal models of AD (Götz et al., 1995). We found that stimulation with glutamate triggers a 4-fold increase in somatodendritic tau that is hyperphosphorylated at an AD-relevant epitope (Braak et al., 1994) (detected by AT8 antibody) (Fig. 4a-c). As shown in Fig. 4c (arrowheads), glutamate also stimulated AT8-immunoreactivity in dendritic spines; these effects were sensitive to the AMPA and NMDA receptor antagonists NBQX and MK801 and were also blocked in the presence of LiCl (Fig. 4c). The last indicates a mediatory role for glycogen synthase kinase-3β (GSK-3β), a major tau kinase. Synaptosomal fractions from glutamate-treated hippocampal neuronal cultures also displayed increased AT8 immunoreactivity (Supplemental Fig. 6).

## 4 Discussion

Tau has two distinct roles: while it is well known to contribute to axonal structure by maintaining microtubule stability, it has recently been recognized to be important in the regulation of synaptic function; specifically, although tau-deficient mice do not display neurostructural abnormalities during development, they cannot express long term depression (LTD), a finding that suggests a role for tau in synaptic function (Kimura et al., 2013). In general, synaptic activity regulates posttranscriptional gene expression by rapidly translating local mRNA (bound to RNA-binding proteins such as Staufen 1 and FMRP, into synaptic proteins (Bramham and Wells, 2007). In this study, tau mRNA as well as CaMKIIα mRNA were found to be associated with FMRP and Staufen1, proteins previously shown to be involved in learning and memory processes that are critically dependent on synaptic function (Lebeau et al., 2008, Sidorov et al., 2013). We found in this study that both, CaMKIIα mRNA and tau mRNA are associated with FMRP, Staufen 1 and myosin Va, and that the translation of these mRNAs were upregulated after stimulation of glutamate receptors. Previous authors reported that glutamate activates CaMKIIα to induce synaptic plasticity (Miller et al., 2002, Coultrap et al., 2014), and that activity-dependent increases in tau protein in excitatory synpases (Frandemiche et al., 2014) represents a physiological mechanism that contributes to synaptic plasticity (Kimura et al., 2013). Notably, tau protein is detectable in postsynaptic terminals in AD brains (Tai et al., 2014); this finding suggests that high AMPA and NMDA receptor stimulation can trigger the aberrant local translation of tau to induce AD pathology. Stress granules are likely to participate in dendritic tau-triggered neurodegeneration since tau is known to interact with the RNA-binding protein T-cell intracellular antigen 1 (TIA1) in stress granules (Vanderweyde et al., 2016).

Tau-triggered neuronal cell death is closely linked with excessive glutamatergic stimulation (Amadoro et al., 2006, Tackenberg et al., 2013), and glutamate itself is implicated in AD-type neurodegeneration (Mattson, 2003). Neither sarcosyl-insoluble tau nor neuronal survival was increased by transitory (5 min) exposure of hippocampal neurons to glutamate (survival monitored over 24 h) in the present study. However, since the treatment paradigm used was sufficient to initiate early processes considered crucial to tau-induced toxicity (increased tau expression, and accumulation of AT8-reactive phosphorylated tau in the somatodendritic compartment), we are currently examining the long-term consequences of single and repeated bursts of AMPA and NMDA receptor stimulation on the formation of insoluble tau in neurons. Although tedious, the information derived from such studies are potentially important given that the risk for AD is increased by factors (*e.g.* traumatic injury (Fleminger et al., 2003), ischemic injury (Biegon et al., 2004), and stroke (Chi et al., 2013) that evoke exaggerated NMDA receptor stimulation. Lastly, the view that excessive neuronal activation may eventually increase neurofibrillary lesions is supported by a very recent report that neuronal activity enhances tau propagation and tau pathology (Wu et al., 2016).

In summary, we have demonstrated that AMPA and NMDA receptor-mediated glutamatergic activation of hippocampal neurons sets off a chain of cellular events that resemble some of those seen in AD pathology (Uchihara et al., 2001, Götz et al., 1995). Briefly, sub-toxic levels of glutamate stimulate the local translation of tau protein from tau mRNA within mRNP granules in the somatodendritic compartment; here, tau accumulates and becomes hyperphosphorylated at epitopes that can potentially result in the formation of neuropil threads and tangles (Braak et al., 1994). These findings are consistent with a recent report (Wu et al., 2016) that neuronal activity can lead to tau pathology. Importantly, they resolve the question of how tau locates to dendrites to induce neurodegenerative processes.

The following are the supplementary data related to this article.

Supplemental information Supplementary Table 1

## Funding Sources

This work was supported by Mext Grant–in-aid project, Scientific Research on Innovation Area, (Brain Protein Aging and Dementia control (to A.T.)), and AMED, Research and development for dementia (to A. T.).

## Conflict of Interest

There are no conflicts of interest to this study.

## Author Contributions

S.K. and T.T. designed, and performed experiments. Y.S. prepared and provided animals. O.F.X.A. provided critical discussion and edited the manuscript. A.T. designed experiments and wrote the manuscript.
