# Supplementary material for: Local Somatodendritic Translation and Hyperphosphorylation of Tau Protein Triggered by AMPA and NMDA Receptor Stimulation
Source: eBioMedicine. 2017 May 17;20:120–6. doi: 10.1016/j.ebiom.2017.05.012 (PMC5478209; doi:10.1016/j.ebiom.2017.05.012)

**SUPPLEMENTAL INFORMATION**

**Supplemental Figures**

**
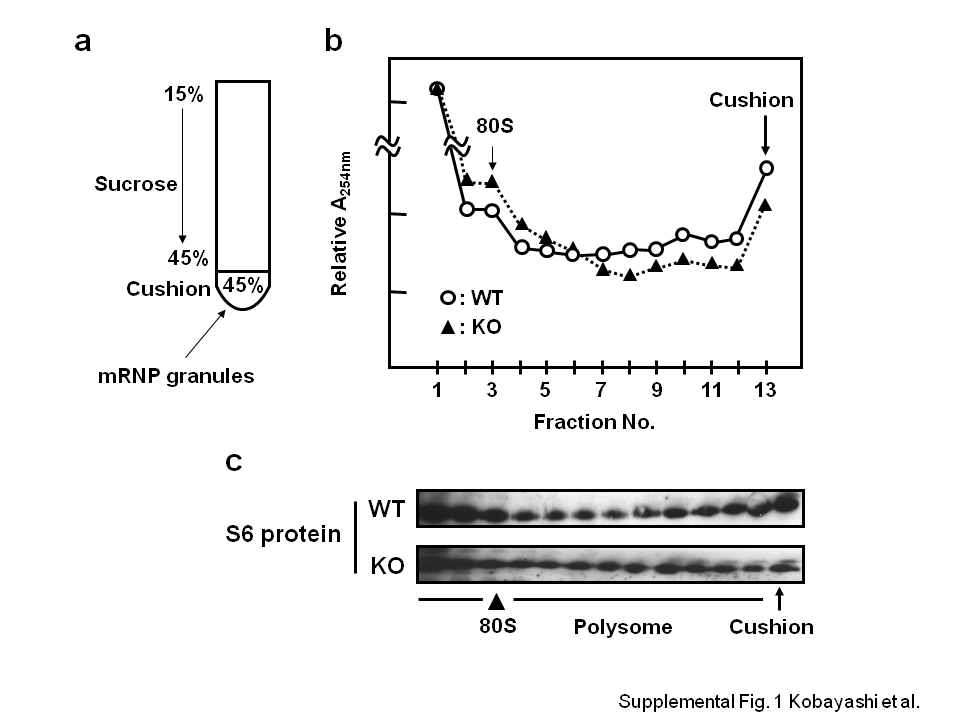
**

**Supplemental Fig. 1. Preparation of the neuronal mRNP granule-enriched (sucrose cushion) fraction.**

**(a)** Schematic representation of the sucrose gradient with cushion. (**b)** Distribution of RNAs in each fraction was monitored by absorbance at 254 nm. WT: wild type mice. KO: tau-knockout mice. (**c)** Distribution of ribosomal protein in each fraction was monitored by detection of S6 ribosome protein by Western blotting.


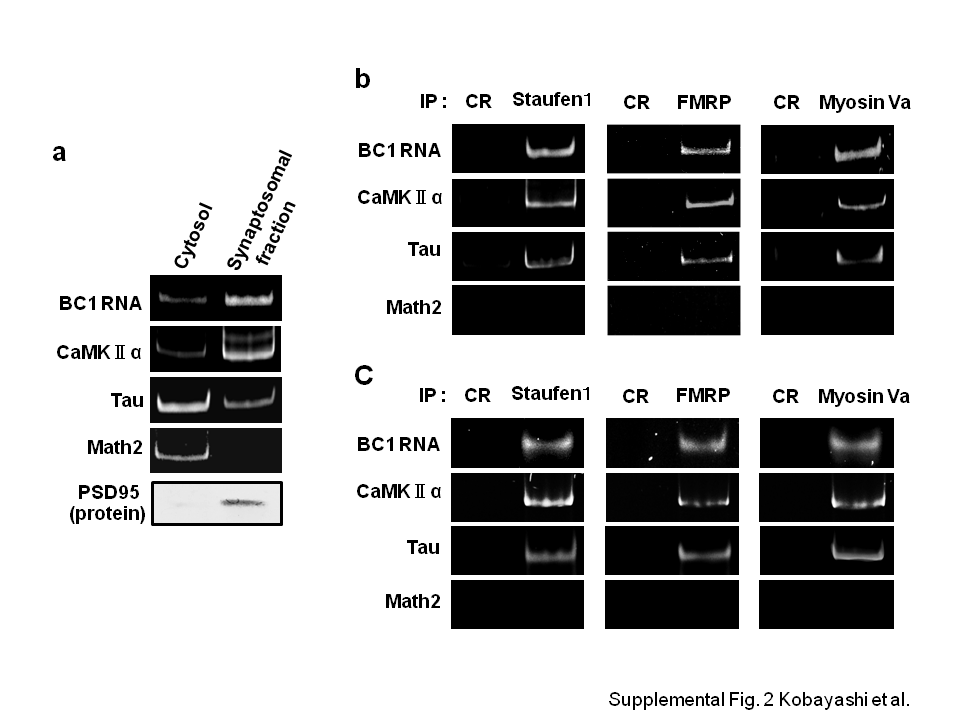


**Supplemental Fig. 2. Synaptosomal ribonucleoprotein (RNP) granules include tau mRNA associated with Staufen1, FMRP and Myosin Va.**

(**a**) Synaptosome fractions were purified from wild type mouse brain. Purity of synaptosomal preparations was confirmed by presence of the post-synaptic protein PSD95 (Western blot) and absence of mRNA encoding the brain-specific transcription factor Math2; as expected, expression of Math2 was restricted to the cytosol. Synaptosomal fractions expressed mRNAs encoding tau, BC1 (small non-coding RNA) and CaMKII α, but not Math2, demonstrating that tau mRNA is present in dendrites and synapses. (**b**) RT-PCR analysis of Staufen1 and FMRP immunoprecipitates of synaptosome fractions from the hippocampi of wild type mice demonstrated the association of these RNP proteins with tau mRNA, as well as CaMKIIα mRNA and BC1. In addition, the mRNAs for tau, CaMKIIα and BC1 were detected in Myosin Va immunoprecipitates as well as in Staufen1, FMRP from spine-enriched synaptosomal fractions. (**c**) The analysis described in (b) was repeated in wild type mice aged 34 weeks; similar results to those found in 5-week old animals were obtained.


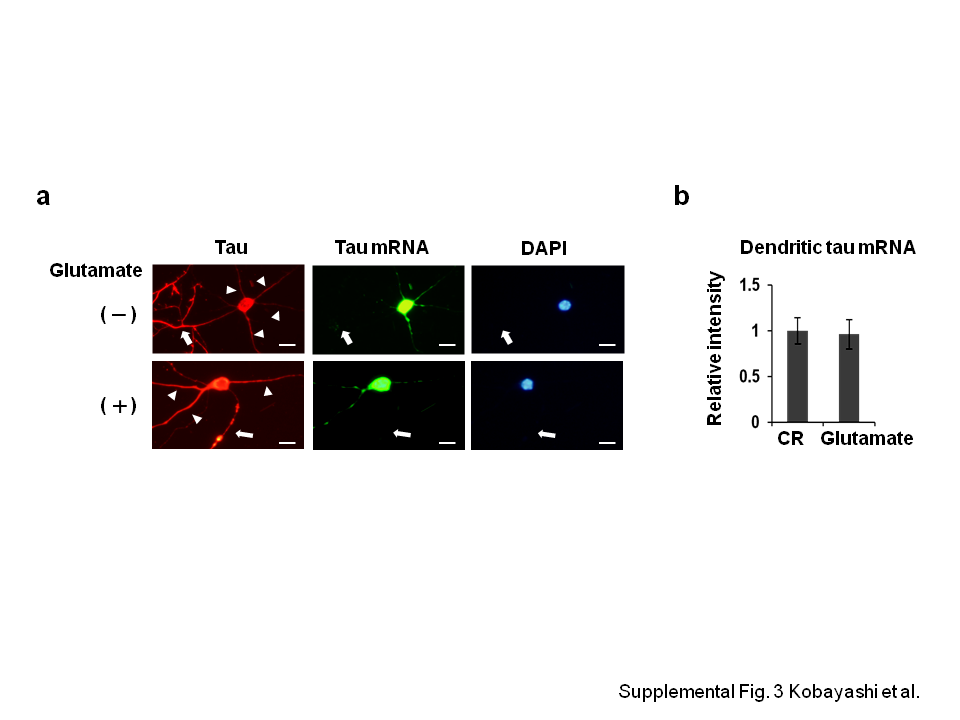


**Supplemental Fig. 3. Glutamate does not stimulate tau transcription.**

Experiments were done in primary hippocampal cultures (a). Fluorescence *in situ* hybridization was used to detect tau mRNA; tau protein was detected by immunostaining (visualized with Alexa Fluor 555); nuclei stained with DAPI. *Upper panel*: control neuron; *lower panel*: neuron treated with glutamate (0.5 mM). *Arrows* and *arrowheads* indicate axons and dendrites, respectively. Scale bar: 10 µm. To quantify levels of tau mRNA (b), signal intensities obtained for approximately 30 dendrites (glutamate-treated or control neurons) using NIH ImageJ software package were normalized to fluorescence intensity of each corresponding DAPI-stained neuron. Tau mRNA levels were not significantly changed by exposure to glutamate.

**
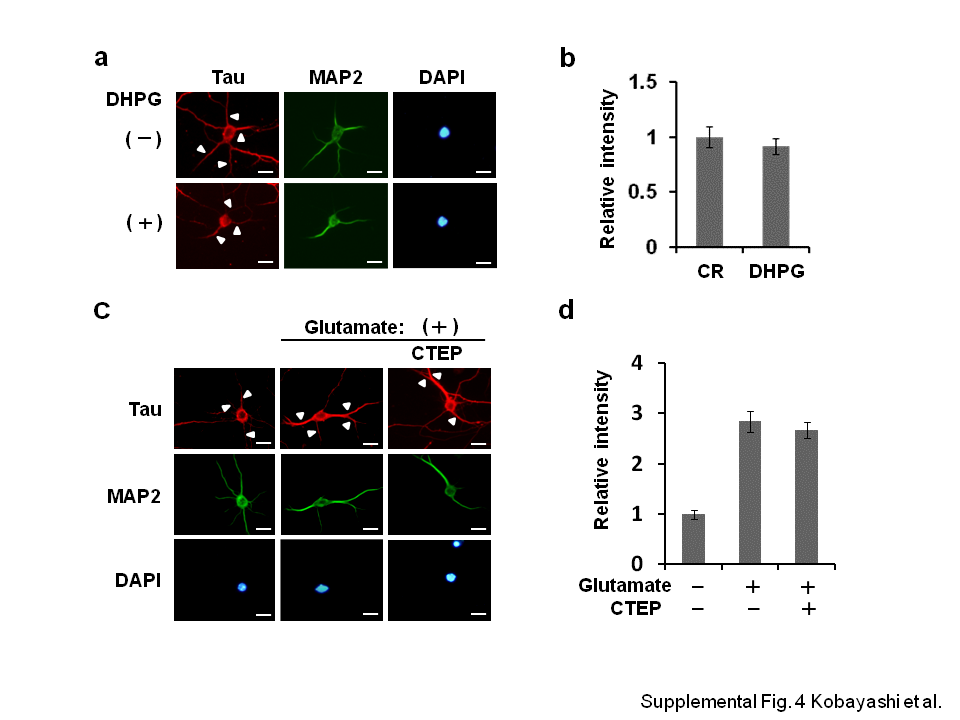
**

**Supplemental Fig. 4. Glutamate-induced translation of dendritic tau mRNA is not mediated by metabotropic glutamate receptors.** To examine whether metabotropic receptors mediate the stimulatory actions of glutamate on tau protein synthesis, glutamate-treated primary hippocampal cultures were co-treated with either DHPG (potent agonist of the group I metabotropic glutamate receptors mGluR1 and mGluR5; 0.5 mM, 5 min) **(a, b)** or with the metabotropic glutamate receptor antagonist CTEP (inhibitor of mGluR; 10 µM) **(c, d)**. Cells were immunostained for tau (visualized with Alexa Fluor 555) and MAP2 (visualized with Alexa Fluor 488) 25 min after treatments. *Arrowheads* indicate dendrites. Data in (**b), (d)** was obtained from fluorescence intensities measured using Image J software (approximately 50 dendrites for treatment sub-groups) which were normalized to the intensity of nuclear DAPI signal. Numerical data represent mean + SEM. Scale bar: 10 µm.

**
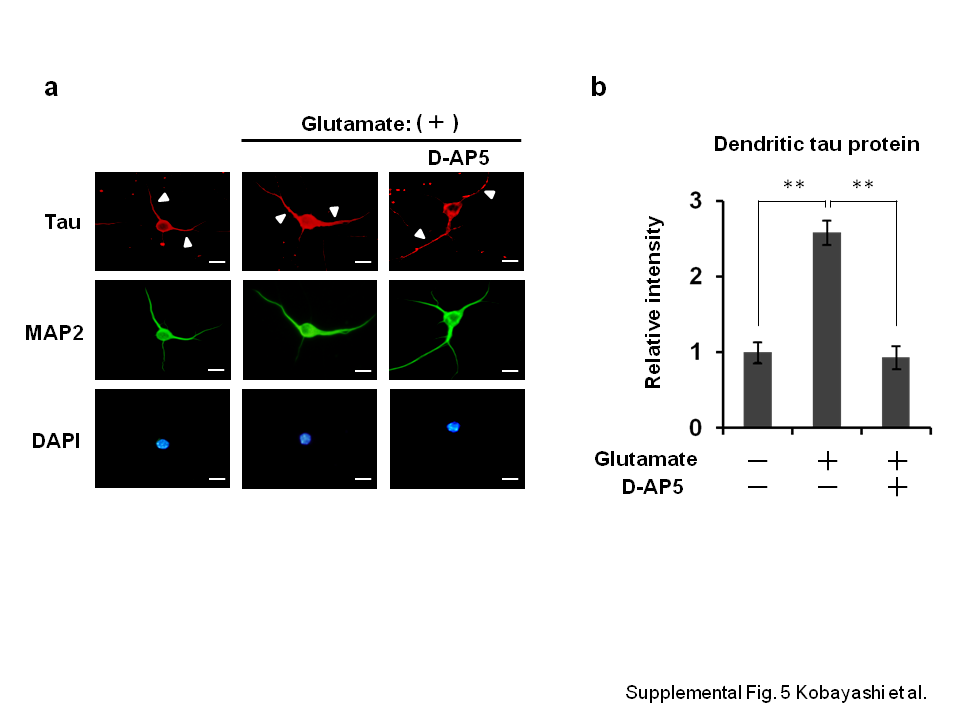
Supplemental Fig. 5. Glutamate-induced translation of dendritic tau mRNA is inhibited by NMDA receptor inhibitor D-AP5**

Glutamate (0.5 mM) effects were blocked by D-AP5 (100 μM), an antagonist of metabotropic NMDA receptors **(a)**. Quantitative analysis of tau (Alexa Fluor 555) immunoreactivity is shown in panels (**b)** as relative tau protein levels (fluorescence intensity) in dendrites in glutamate-treated and untreated control (CR) hippocampal neurons; for this, approximately 25 neurons were evaluated. Data, expressed as mean + SEM, were normalized to intensity of nuclear DAPI staining. **P <0.01 (Student’s *t* test). Scale bar: 10 µm**.**


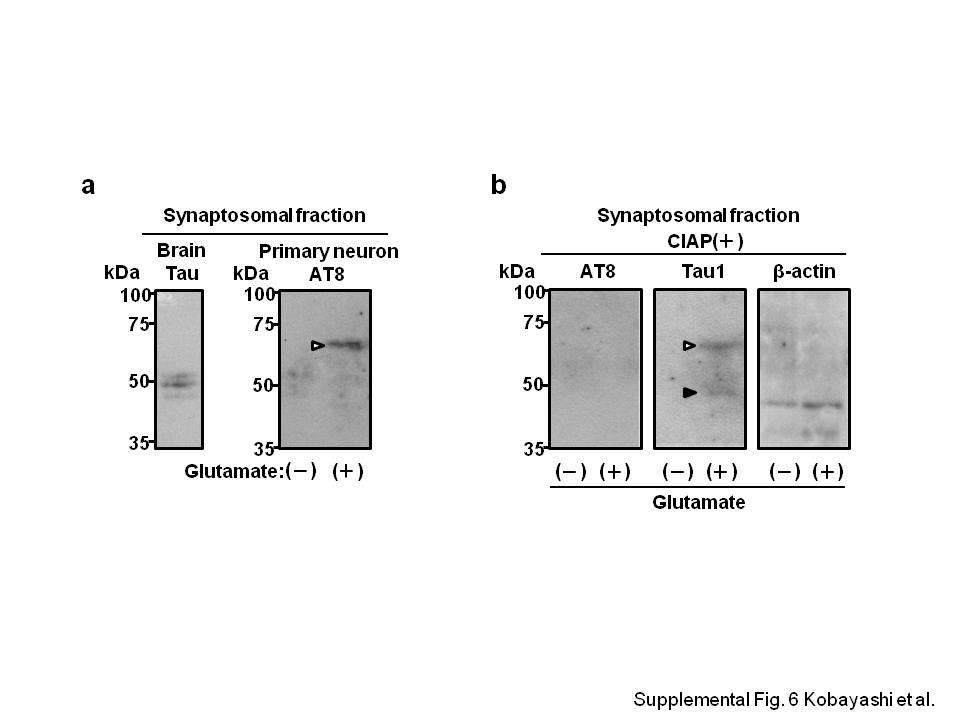


**Supplemental Fig. 6.**

**(a)** Synaptosomal fractions from glutamate-treated or untreated cells were analyzed by Western blotting using anti-AT8 antibody (*right panel*). Synaptosomal preparations were verified as before (Supplementary Fig. 2). Wild-type mouse brain, shown to express tau protein (immunoblotting) served as control (Brain; *left panel*). **(b)** After stripping the AT8 antibody, membranes were incubated with alkaline phosphatase (CIAP) at 37℃ for 2 h, and the AT8 and Tau1 immunoreactivities (*left and central panels*) visualized. After another stripping, membranes were used to detect β-actin (*right panel*). *Open arrowhead*: AT8-positive signal in (**a)**, and Tau1-positive signal in (**b)**. *Closed arrowhead*: Tau1 positive signal. Note that the spine-enriched fractions from glutamate-treated cells displayed an AT8 immunoreactive band (68 kDa) but that no signal was detectable in preparations from untreated cells **(a);** native tau has a size of ~50 kDa. Confirmation that glutamate upregulates the accumulation of highly phosphorylated tau was provided by the observation that treatment of the membrane with calf intestinal alkaline phosphatase (CIAP) abolished the 68 kDa AT8-positive band **(b)** to yield Tau1-immunoreactive bands of non-phosphorylated tau at 68 and 50 kDa.

**Supplemental Table 1.**

**Primer pairs used for RT-PCR.**


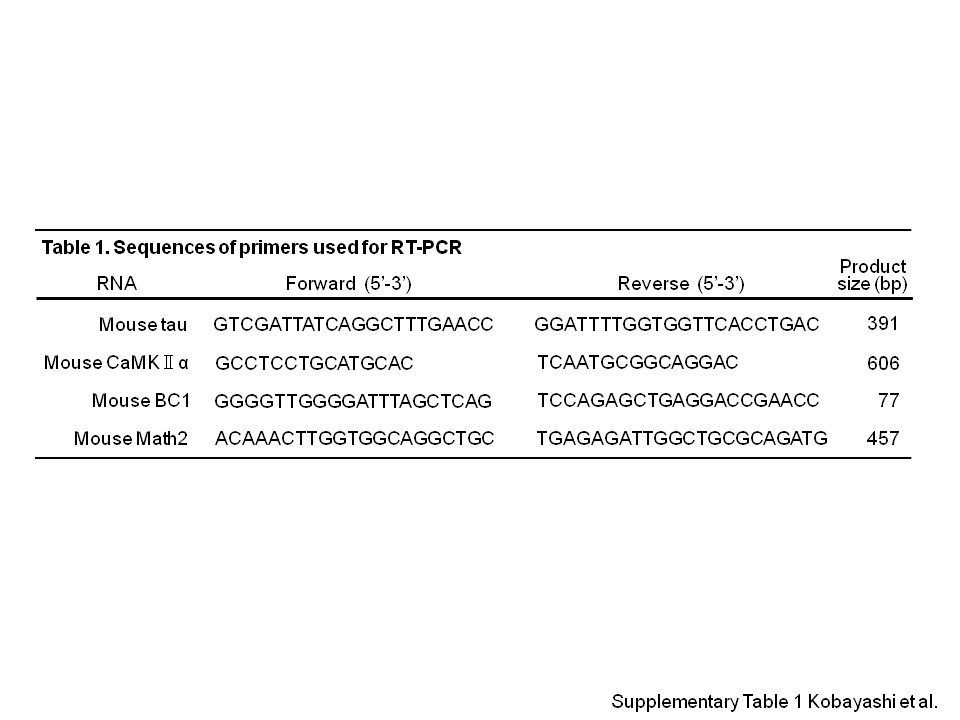


Nucleotide sequences and product sizes are indicated.


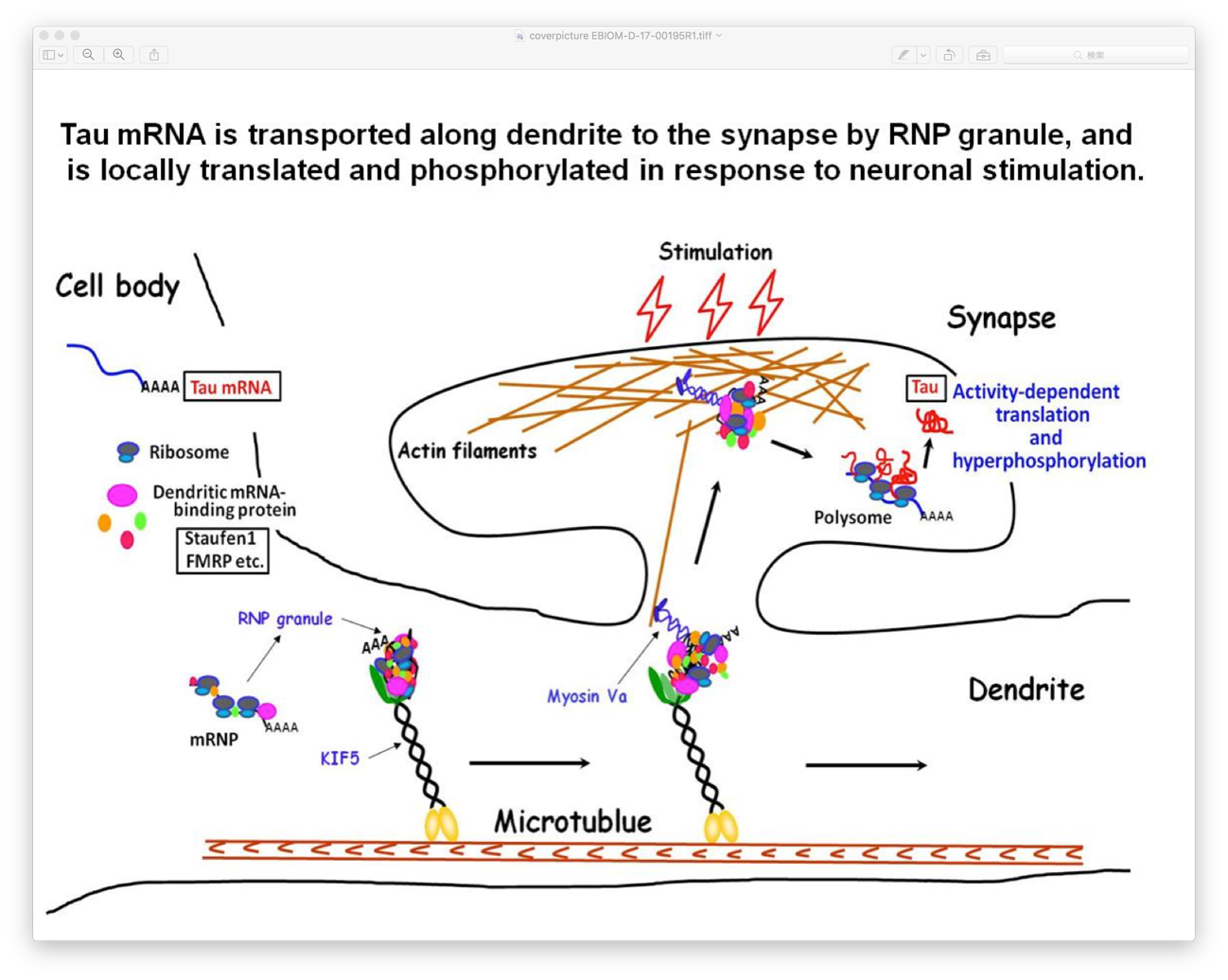

Supplement: Supplementary file 1 — Supplemental information [file mmc1.docx]
